# The utility of multi-stack alignment and 3D longitudinal image registration to assess bone remodeling in rheumatoid arthritis patients from second generation HR-pQCT scans

**DOI:** 10.1186/s12880-020-00437-8

**Published:** 2020-04-07

**Authors:** Scott C. Brunet, Michael T. Kuczynski, Jennifer L. Bhatla, Sophie Lemay, Yves Pauchard, Peter Salat, Cheryl Barnabe, Sarah L. Manske

**Affiliations:** 1 Department of Radiology, Cumming School of Medicine, University of Calgary, Calgary AB3280 Hospital Dr NW, Calgary, Alberta T2N 4Z6 Canada; 2 Biomedical Engineering Graduate Program, Schulich School of Engineering, University of Calgary, Calgary, AB Canada; 3 McCaig Institute for Bone and Joint Health, Cumming School of Medicine, University of Calgary, Calgary, AB Canada; 4 Division of Rheumatology, Department of Medicine, Cumming School of Medicine, University of Calgary, Calgary, AB Canada

**Keywords:** HR-pQCT, Image registration, Rheumatoid arthritis, Bone remodeling

## Abstract

**Background:**

Medical imaging plays an important role in determining the progression of joint damage in rheumatoid arthritis (RA). High resolution peripheral quantitative computed tomography (HR-pQCT) is a sensitive tool capable of evaluating bone microarchitecture and erosions, and 3D rigid image registration can be used to visualize and quantify bone remodeling over time. However, patient motion during image acquisition can cause a “stack shift” artifact resulting in loss of information and reducing the number of erosions that can be analyzed using HR-pQCT. The purpose of this study was to use image registration to improve the number of useable HR-pQCT scans and to apply image-based bone remodeling assessment to the metacarpophalangeal (MCP) joints of RA patients.

**Methods:**

Ten participants with RA completed HR-pQCT scans of the 2nd and 3rd MCP joints at enrolment to the study and at a 6-month follow-up interval. At 6-months, an additional repeat scan was acquired to evaluate reliability. HR-pQCT images were acquired in three individual 1 cm acquisitions (stacks) with a 25% overlap. We completed analysis first using standard evaluation methods, and second with multi-stack registration. We assessed whether additional erosions could be evaluated after multi-stack registration. Bone remodeling analysis was completed using registration and transformation of baseline and follow-up images. We calculated the bone formation and resorption volume fractions with 6-month follow-up, and same-day repositioning as a negative control.

**Results:**

13/57 (23%) of erosions could not be analyzed from raw images due to a stack shift artifact. All erosions could be volumetrically assessed after multi-stack registration. We observed that there was a median bone formation fraction of 2.1% and resorption fraction of 3.8% in RA patients over the course of 6 months. In contrast to the same-day rescan negative control, we observed median bone formation and resorption fractions of 0%.

**Conclusions:**

Multi-stack image registration is a useful tool to improve the number of useable scans when analyzing erosions using HR-pQCT. Further, image registration can be used to longitudinally assess bone remodeling. These methods could be implemented in future studies to provide important pathophysiological information on the progression of bone damage.

## Background

Rheumatoid arthritis (RA) is a chronic, inflammatory, autoimmune disease that affects peripheral joints, such as the metacarpophalangeal (MCP) joints of the hands. In RA, pro-inflammatory cytokines and disease-specific autoantibodies stimulate an increase in osteoclast activity, resulting in local bone resorption and an imbalance in local bone remodeling [1–3]. This results in a loss of bone density and surrounding bone microarchitecture, which in turn causes pathological cortical interruptions known as bone erosions [1, 2]. These periarticular bone erosions are a key outcome measure of RA, and their presence, size, and number are commonly used in diagnosis and monitoring of disease progression [1–4].

While other imaging modalities such as ultrasound, magnetic resonance imaging, and conventional radiography are more common for imaging hand RA, they are unable to detect small erosions due to their low spatial resolution. High-resolution peripheral quantitative computed tomography (HR-pQCT) is a sensitive tool capable of imaging bone microarchitecture, bone density, and erosion size and number with high precision [5–7]. The high spatial resolution provided by HR-pQCT enables the analysis and quantification of small erosions, which are clinically significant as it has been shown that patients with RA have a greater number of small erosions compared to healthy controls [8], and have shown to have an association with pain and decreases in function [9]. Erosion volume has been calculated using manual, semi-automated and fully-automated tools with high reproducibility [10–12].

The standardized scan acquisition protocol for MCP joints of the hand using HR-pQCT to evaluate and quantify joint changes in RA was designed with the primary purpose of quantifying joint space [13]. While this protocol allows for visualization of the joint space, metacarpal head, and proximal phalanx, current limitations in the technology require the acquisition to be conducted in three sequential “stacks” to form a three-dimensional (3D) model of the MCP joints. Patient movement during or between the stack acquisition can cause misalignments between stacks, termed “stack shift” artifacts. These artifacts may increase the difficulty in analyzing bone erosions that span two stacks by causing a misalignment, thereby impacting determination of the erosion volume, and potentially impacting the assessment of the presence of an erosion. Prior studies have addressed this issue by only analyzing erosions contained within a single stack, or by discarding scans with stack shift artifacts [14–16]. In a study that proposes an automated method for quantifying longitudinal changes in erosions using 3D registration, 43% of HR-pQCT scans of the MCP joints had to be excluded due to severe motion artifact [6]. To minimize data loss, the assessment of all bone erosions is necessary.

As well as measuring erosion presence and volume in a single scan, rigid 3D image registration is a technique that can be used to visualize and quantify longitudinal changes in erosion size and shape by monitoring changes in cortical and trabecular bone [6]. Tracking these bone changes longitudinally aids in determining if bone healing or further damage is occurring. Further, bone remodeling in an entire region of interest can be visualized and quantified [17, 18]. However, the accuracy of this image registration technique is reduced when stack shift artifacts are present. To our knowledge, voxel-based bone remodeling has not been implemented on scans from a second-generation HR-pQCT scanner.

The purpose of this study was to use 3D rigid image registration to reduce the impact of stack artifact on visualization and quantification of bone changes in the HR-pQCT images of the MCP joints of patients with RA. We propose a new HR-pQCT scanning protocol for the 2nd and 3rd MCP joints and a semi-automated image registration method to align the stacks and reduce the impact of stack shift artifact. This in turn allows for a more robust method to longitudinally assess bone erosions and bone remodeling. Further, we applied a 3D longitudinal image registration and bone remodeling analysis technique and adapted it to the higher resolution second generation HR-pQCT scanner. The sensitivity of these analyses to motion and interpolation method were evaluated. These image registration techniques could be important tools in analyzing bone changes over time as seen on HR-pQCT for patients with RA.

## Methods

### Participants

Ten participants with RA in physician-classified remission were recruited using the Rheum4U platform [19] from the Division of Rheumatology at the University of Calgary. In order to be included in the study, participants had to be over the age of 18 and meet the American College of Rheumatology/European League against Rheumatism 2010 Classification criteria for rheumatoid arthritis diagnosis [20]. Participants with a history of MCP injury, replacement, or whose conventional radiographs showed no joint space in the MCPs were excluded.

### HR-pQCT image acquisition and analysis

HR-pQCT scans of the 2nd and 3rd MCP joints of the participants’ hand were acquired (XtremeCTII, Scanco Medical, Brüttisellen, Switzerland) at baseline and at a 6-month follow-up. Quality control scans were performed daily using a phantom with rods of known density. A same-day repeat scan after repositioning was obtained at the follow-up visit. The hand was secured in a custom positioning device and used an adaptation of an established protocol [21] to reduce the impact of motion on image processing. The scan was acquired with a nominal isotropic resolution of 60.7 μm using the manufacturer standard settings (68 kVp, 1470 μA, 43 ms integration time). We obtained a reference x-ray in the coronal plane, and a reference line was placed at the distal cortical surface of the 2nd metacarpal head. Three 10.2 mm sections or “stacks” were acquired separately with a 25% (2.55 mm) overlap of the previous stack resulting in a total scan length of 25.5 mm. A 25% overlap was selected after testing because it allowed for consistent registration results, while maintaining an adequate scan distance to include both the proximal phalange and distal metacarpal bones across both the 2nd and 3rd MCPs. Each stack was evaluated for motion using the manufacturer’s standard scoring system from 1 to 5 [22]. Data that included a motion score of 4 or 5 were excluded from the longitudinal analysis and scan-rescan analysis. Erosions were identified by a trained reader. Image processing was completed using Image Processing Language (IPL v5.42, Scanco Medical). The analysis scripts generated and used during this study will be made available through the Manske Lab GitHub repository.

### Multi-stack registration

**Fig. 1:**
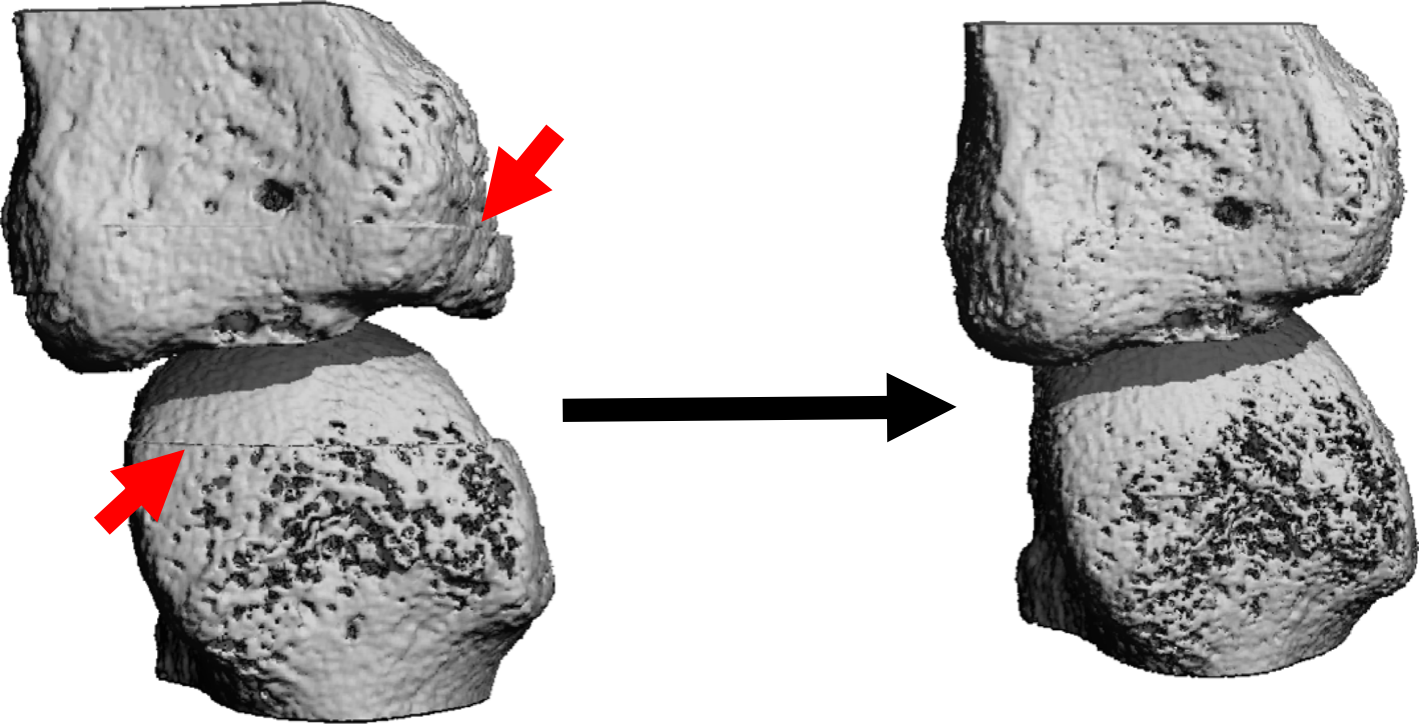
An example of a joint that was scanned and aligned without registration (left) and with multi-stack registration (right). Red arrows indicate the stack shift artifact suggesting changes in the average position between the stacks

The proximal, middle, and distal stacks of the 2nd and 3rd MCP joints were registered in order to achieve optimal alignment. Each stack was registered using the 25% overlap of the previous stack. The registration was initialized using the mass centers and moment of inertia to give an estimate of the new orientation. Once the registration was initialized, the proximal stack was registered to the middle stack using a simplex optimization to determine rigid transformation parameters by maximizing the correlation coefficient. The proximal stack was then transformed to the middle stack image space using a cubic interpolator. The same procedure was followed to register the distal stack to the middle stack. Masks of the overlapped regions were created and used to remove the common regions with the middle stack from the distal and proximal stacks. Finally, the transformed proximal and distal stacks (excluding the overlapped region) were concatenated to the middle stack (including the overlapped region) creating an image of the three stacks optimally aligned. The final image of the joint includes the proximal and distal stacks registered, transformed, and aligned with the middle stack to give the appearance of contiguous bone, with the stack shift artifact eliminated (Fig. 1).

### Longitudinal image registration

In order to investigate bone damage and healing phenomena in RA, longitudinal 3D rigid body image registration was implemented to align and superimpose the follow-up image with the baseline image. The first step in the image registration workflow was to determine a transformation matrix to rotate and translate the follow-up image to the baseline image space. For the purpose of this registration, the periosteal mask of either the metacarpal or phalange bone was dilated and then used to crop the desired bone, with a 5-voxel border of background. From there, the center of mass and moment of inertia was used to initialize the registration. This rigid registration used a simplex optimizer approach and a correlation object function to iteratively find the optimal fit between the follow-up and the baseline image. The transformation matrix was then applied to the follow-up image to create a translated and rotated image that was resampled using cubic interpolation. Finally, the images were masked within the largest common volume (LCV) to exclude any voxels outside the common region.

### Bone remodeling analysis

**Fig. 2:**
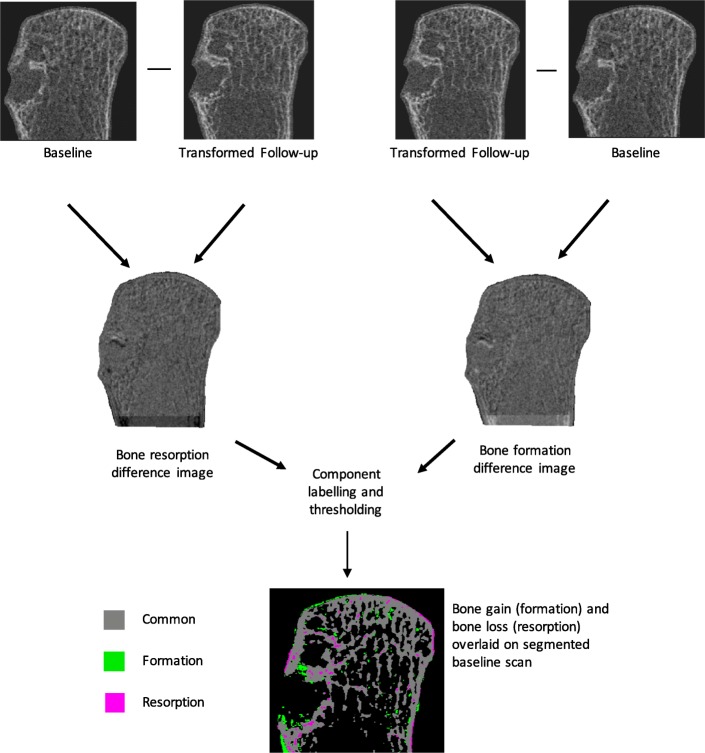
The workflow for the bone remodeling analysis. After registration, the transformed follow-up image is subtracted from the baseline image to form a bone resorption difference image. The baseline image is subtracted from the transformed follow-up image to for a bone formation difference image. These difference images are then segmented at a threshold of 125 mgHA/cm^3^ and overlaid on the segmented baseline image to form a comprehensive image visualizing bone formation and resorption

Once the baseline and follow-up images were aligned in the same image space, they were analyzed to find regions of bone formation and bone loss. This was accomplished by subtracting the grey-scale density images from one another voxel-by-voxel within the LCV based on a method reported previously [18]. The resulting image therefore represents the differences in densities between the two images, which can represent areas of local bone remodeling. The follow-up image was subtracted from the baseline image and then segmented with a threshold value of 125 mgHA/cm^3^ so that any voxels above this threshold would represent bone resorption. The baseline image was subtracted from the follow-up image and then segmented so that any voxels greater than the threshold value of 125 mgHA/cm^3^ would represent bone formation. To eliminate noise from the difference image, a connected components filter was then applied so that clusters of voxels connected by fewer than 5 voxels were removed from the image. These segmented regions of bone loss and bone gain were then overlaid with the segmented baseline scan to create a comprehensive image depicting bone loss and bone gain (Fig. 2). The threshold and connected components cluster size was determined based on previous work [17, 18], and adapted for the second generation HR-pQCT scanner and MCP joints to provide a plausible depiction of bone formation and bone resorption based on visual inspection of the grey-scale images at baseline and follow-up as well as the final segmented image. Bone formation and resorption fractions were then quantified as a percentage of the total voxels labelled as formation or resorption from the respective difference image. These formation and resorption fractions were also calculated using the same bone remodeling script for the same-day scan-rescan after repositioning. This was used as a negative control to ensure that any changes observed over the 6-months were due to bone change rather than repositioning in the scanner or image artifact.

### Effects of image interpolation on bone remodeling assessment

After registering the follow-up (moving) image to the baseline (fixed) image, the moving image was transformed to the fixed image space. Following this transformation, cubic interpolation was performed to realign the moving image to the fixed image’s coordinate system. Image interpolation can be seen as a linear convolution with a low-pass filter, and therefore, may have an effect on bone morphometric analysis [23, 24]. To analyze the effects of interpolation on the bone remodeling analysis, the bone remodeling values were compared for registration of the 6-month follow-up scans to their respective same-day rescans where both scans underwent a cubic interpolation and where only the moving image underwent interpolation.

### Statistical analysis

Descriptive statistics were used to observe if there were any improvements in the number of erosions that could be analyzed with multi-stack registration, compared to scans without multi-stack registration. Kruskal-Wallis tests were used as a non-parametric method to compare the differences in bone formation and resorption fraction across different motion grades against an alpha value of 0.05. A Wilcoxon signed rank test was used to compare the difference in bone formation and resorption fraction between registrations with only the moving image interpolated compared to registrations where both scans undergo interpolation against an alpha value of 0.05. Results are reported as median (interquartile range, IQR) unless otherwise stated. Analysis was completed using R (v3.4.3) in RStudio (v1.1.423).

## Results

### Stack shift artifact

**Fig. 3:**
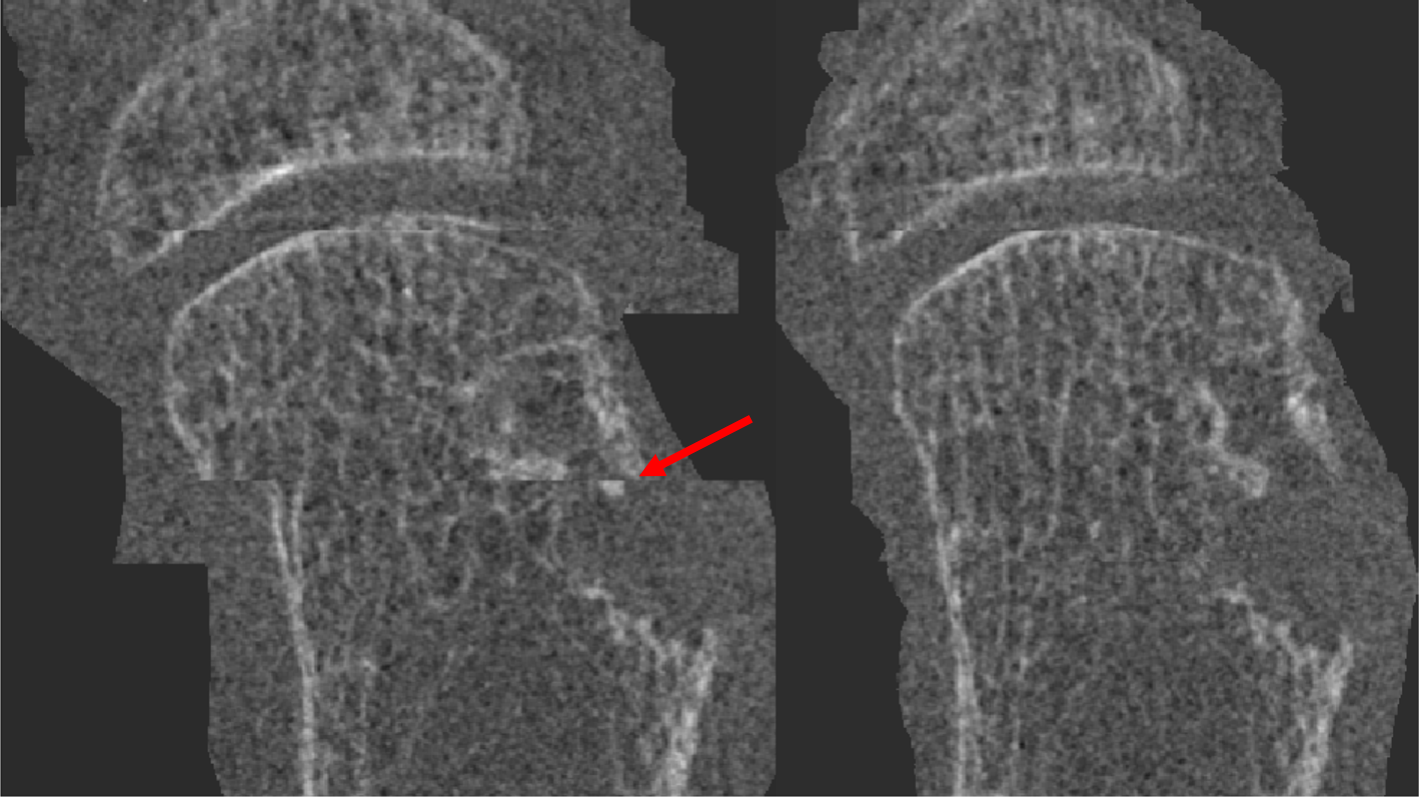
An example of an erosion that would not have been quantifiable without stack realignment (left) compared to the same scan with stack registration to correct for the stack shift artifact (right)

Fifteen joints from 8 participants were analyzed for erosions both using multi-stack registration and without. Two participants were excluded due to motion or acquisition errors preventing successful multi-stack registration at either baseline or follow-up, and an additional joint was excluded due to inaccurate registration. Of the 57 erosions analyzed from baseline and follow-up combined, 13 erosions (23%) could not be analyzed from raw images due to a stack shift artifact. These erosions crossing stack boundaries were present in 8 of the 15 joints (53%). The erosions could be volumetrically assessed after stack registration (Fig. 3).

### Bone remodeling analysis

**Fig. 4:**
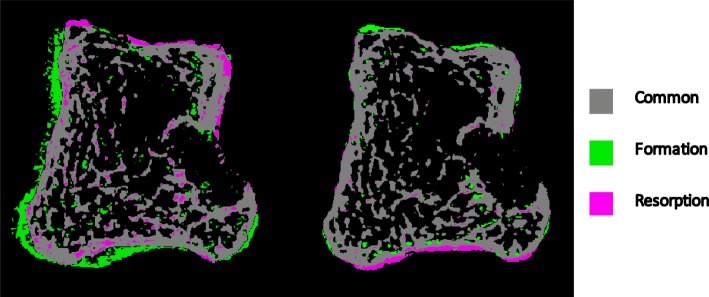
An example of a longitudinal registration between 0 months and 6 months of a 3rd metacarpal bone for a scan with no stack registration (left) and with stack registration (right)

**Fig. 5:**
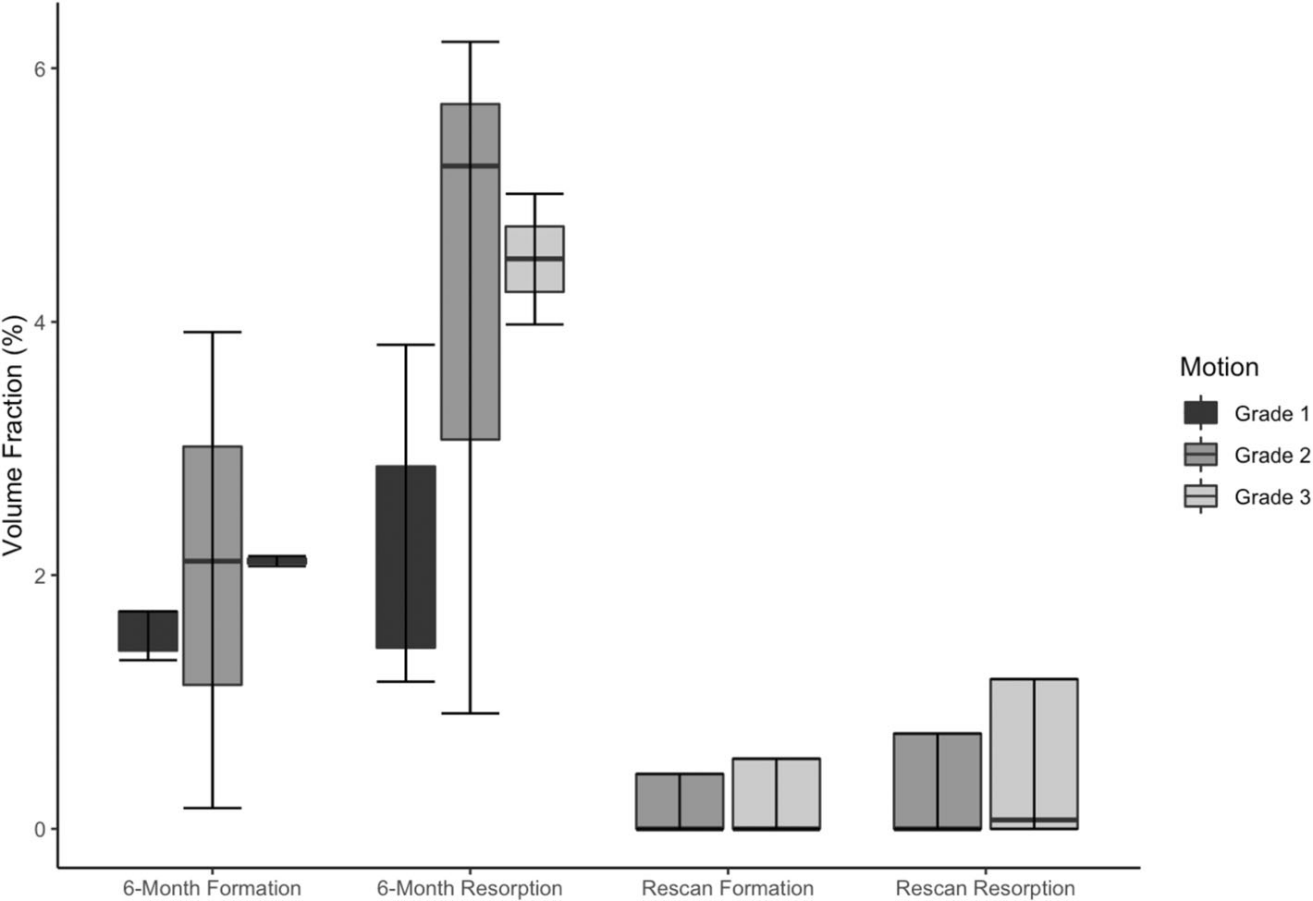
The bone formation volume fraction and bone resorption volume fraction for 6-month longitudinal scans (*n* = 9 joints) and same-day scan-rescan (*n* = 8 joints) for different motion scores. The highest motion score from the two scans was used

Of the 15 joints that were aligned successfully with multi-stack registration, 9 joints from 5 participants were included in bone remodeling analysis as 6 joints did not meet our motion scoring criteria for quantitative assessment, despite having the stacks successfully aligned. Based on visual inspection, multi-stack registration improved our ability to perform longitudinal registration to assess bone remodeling (Fig. 4). The median (and IQR) for bone formation and resorption fractions were 2.1% (IQR 1.4–2.2%) and 3.8% (IQR 1.5–5.0%) respectively over the 6-month follow-up. The median bone formation fraction appeared to increase with an increasing motion score from 1 to 3 (1.5% (IQR 1.4–1.7%), 2.1% (IQR 1.1–3.0%), and 2.1% (IQR 2.1–2.1%) respectively). The mean bone resorption fraction also trended towards an increase with an increasing motion score from 1 to 3, (2.0% (IQR 1.4–2.9%), 5.2% (IQR 3.1–5.7%), and 4.5% (IQR 4.2–4.8%) respectively) (Fig. 5). However, these trends were not statistically significant (*p* > 0.05).

### Scan-rescan reproducibility

To determine the effects of repositioning and inherent noise on image-based bone remodeling metrics, eight joints from 4 participants were analyzed for bone formation and resorption using the 3D registration of 2 scans conducted on the same day after repositioning. One additional participant was excluded due to motion artifact exceeding our criteria in the repeat scan. The median bone formation reported between the same-day rescan was 0.0% (IQR 0.0–0.4%), while the mean bone resorption was 0.0% (IQR 0.0–0.9%). The differences between the 6-month longitudinal bone formation and resorption fractions with the negative control fractions were statistically significant (*p* < 0.05). There was no significant difference (*p* > 0.05) in bone formation fraction with an increasing motion score from 2 to 3 (0.0% (IQR 0.0–0.4%) and 0.0% (IQR 0.0–0.6%) respectively) or bone resorption fraction with an increasing motion score from 2 to 3 (0.0% (IQR 0.0–0.8%) and 0.1% (IQR 0.0–1.2%) (Fig. 5).

Scan-rescan analysis was also performed with a cubic interpolator applied to both the fixed and moving image rather than just the moving image with the intention of analyzing the effects of interpolation on the bone remodeling analysis. There were no significant differences found for either bone formation or bone resorption when comparing this interpolation method with interpolation of the moving image only (*p* > 0.05).

## Discussion

In this study, image registration was successfully used to align multiple stacks in HR-pQCT images of the 2nd and 3rd MCP joints, reducing the impact of stack shift artifact to improve the number of usable scans and increase the number of erosions that could be analyzed longitudinally. Consistent with our current study, where 23% of erosions could not have been quantified without stack registration, we observed in a different cohort of patients that 10/48 (21%) of images had to be excluded from erosion analysis due to stack shift artifact that crossed through the middle of an erosion [25]. Further, a 3D longitudinal image registration and bone remodeling analysis method was applied to these MCP scans. It was observed that over the course of 6 months, there was a net loss of bone in patients classified in clinical remission. Both of these image registration techniques could be implemented in future HR-pQCT studies to quantify bone changes over time in patients with RA.

While multi-stack registration reduces the impact of the stack artifact and improves longitudinal image registration to assess bone remodeling, motion artifact still has an impact on the quantitative analysis of bone remodeling. Although it was not significant, we observed that low quality images of the MCP joints with higher motion grades led to a trend towards higher bone formation and resorption volumes compared with images with a higher quality and lower motion grades. This trend was also observed in a previous study using a similar bone remodeling analysis method on the radius and tibia on the first generation HR-pQCT scanner [18]. This suggests that even if scans meet the motion scoring criteria to be included in analysis, motion could still have an impact on bone remodeling analysis. Despite the improvements in the number of erosions available for analysis after multi-stack registration, there were several stacks with a high motion grade that had to be excluded from the study. Further, visual inspection was used to confirm registration alignment. Using rigid registration, we presumed that no significant changes in shape occurred over the 6-month period. In particular, our visual inspection focused primarily on the region around the erosion as this was our primary region of interest. Slight errors in registration and/or motion artifact may lead to inaccuracies in the global bone remodeling analysis. Previous studies using microCT in animal models have demonstrated that longitudinal analyses of bone remodeling produces better accuracy and precision when performed on a local region of interest rather than the whole bone [26–28]. It is likely that future studies using this bone remodeling analysis in the context of rheumatoid arthritis will be primarily concerned with bone changes around the region of an erosion, rather than the global changes throughout the bone. Therefore, in addition, further methods to reduce motion artifact and improve image registration, including focus on a local region of interest, should be investigated. In addition, future studies in rheumatoid arthritis should evaluate the utility of bone remodeling analysis in the peri-erosion region.

We also explored whether applying interpolation to both fixed and moving images decreased the interpolation error to an extent that it impacted bone remodeling volume fraction. The application of a transformation matrix to the moving image results in errors caused by image interpolation which may have an impact on longitudinal bone analysis [24]. However, by comparing bone remodeling fractions between scan-rescan registrations where a cubic interpolation was applied to either just the moving image or both images, we found that the difference in bone remodeling fractions due to interpolation was not significant. These results are consistent with previous work comparing the effects of various image interpolation methods on bone morphometric analysis from micro-CT scans using an animal model [24]. The current study demonstrated this finding in a human population using HR-pQCT scans.

Scan-rescan analysis was also completed to act as a negative control. There was a significant difference in the bone formation and resorption fractions between the 6-month longitudinal and negative control, indicating that the changes observed in formation and resorption were due to actual bone changes, rather than image noise and patient repositioning. Part of the challenge for scan-rescan analysis of bone remodeling may be due to the absence of scans with a motion score of 1 (i.e., no motion). As a result, the scan-rescan bone remodeling volume fractions may have been overestimated when compared to the longitudinal analysis based on the lack of any extremely high-quality scans. While variations in x-ray tube or detector functioning can affect the density values used to perform bone remodelling analysis, the fluctuations recorded on the daily quality control scans were very small relative to the differences detected over the 6-month follow-up period.

There are some limitations associated with the multi-stack image registration technique. First, the acquisition of 3 separate stacks can be challenging for the operator and participant. As an overlapping acquisition protocol has not been implemented by the manufacturer, each stack must be acquired individually rather than contiguously. This results in a longer acquisition time, as well as the scanner stopping and starting between stacks, which could potentially lead to even more movement. This may also explain why there was such a large proportion of scans that would have had to be excluded without the registration of stacks as the noticeable breaks between stack acquisition are not present in the standard protocol. Further, this technique does not eliminate motion artifact, as the reconstruction of each stack takes into account the average position of each stack. When there is motion within a stack, this can impact the scan in a way that cannot be corrected by this image registration technique. While the current study improved the number of scans that could be analyzed, only 9 out of 20 joints (45%) met the criteria to be included in the longitudinal analysis. There were 4 joints that could not be analyzed due to acquisition error or motion that prevented successful multi-stack registration, 1 joint that failed stack registration despite appropriate motion scores, and 6 joints that successfully had the stacks aligned, but were excluded since they did not meet our motion criteria. Despite the benefits of the multi-stack registration, motion artifact still presents a major limitation when analyzing HR-pQCT scans of the MCP joints. Future investigations should build upon this work to reduce the impact motion artifact has on these scans.

There are also limitations associated with the bone remodeling analysis method used in this study. First, the threshold value of 225 mgHA/cm^3^ presented in previous work [17, 18] was not used in this study. After several tests assessing the accuracy of the results with visual inspection, it was observed that obvious areas of bone changes on the grey-scale images were not detected using this threshold for the RA patients in this cohort. Therefore, a new threshold was selected (125 mgHA/cm^3^) after several tests that appeared to give the best depiction of bone change. This may be because the previous work was done on the radius and tibia, on the first-generation HR-pQCT scanner with lower spatial resolution, which may have different thresholds of change than the MCP joints analyzed in the current study. This new threshold should be validated using cadaveric samples to ensure that the change being visualized is due to real bone change and not noise.

The multi-stack image registration technique proposed in this study could be a valuable tool to improve the utilization of HR-pQCT scans for the research of bone changes in RA. Improving the number of useable scans will be instrumental in furthering the use of HR-pQCT to study bone changes in RA. Further, 3D longitudinal image registration can be applied to second generation HR-pQCT scans to visualize and quantify bone changes at a higher resolution, over a relatively short time period (6-months) without the need for a bone biopsy.

## Conclusions

Multi-stack imaging can be used to increase coverage of HR-pQCT scans. The multi-stack registration tool presented here is useful to decrease the effects of motion on multi-stacks. This tool can improve the number of useable scans when analyzing erosions and other pathological defects, as well as to longitudinally assess bone remodeling in larger regions of interest. These methods could be implemented in future studies to provide important pathophysiological information on the progression of bone damage.

## Supplementary information

**Additional file 1.**

## Data Availability

The datasets generated during this study are included in this published article and available as supplementary material. The analysis scripts generated and used during this study will be made available through the Manske Lab GitHub repository at https://github.com/ManskeLab/XCT_IPL_Registration.

## Abbreviations

3D: Three-dimensional
HR-pQCT: High-resolution peripheral quantitative computed tomography
IPL: Image Processing Language
IQR: Interquartile Range
LCV: Largest Common Volume
MCP: Metacarpophalangeal
RA: Rheumatoid arthritis
